# Recent insights into the tick microbiome gained through next-generation sequencing

**DOI:** 10.1186/s13071-017-2550-5

**Published:** 2018-01-04

**Authors:** Telleasha L. Greay, Alexander W. Gofton, Andrea Paparini, Una M. Ryan, Charlotte L. Oskam, Peter J. Irwin

**Affiliations:** Vector and Waterborne Pathogens Research Group, School of Veterinary and Life Sciences, Murdoch University, Perth, WA Australia

**Keywords:** Ticks, Microbiome, Next-generation sequencing, Bacteria, Protozoa, Viruses

## Abstract

The tick microbiome comprises communities of microorganisms, including viruses, bacteria and eukaryotes, and is being elucidated through modern molecular techniques. The advent of next-generation sequencing (NGS) technologies has enabled the genes and genomes within these microbial communities to be explored in a rapid and cost-effective manner. The advantages of using NGS to investigate microbiomes surpass the traditional non-molecular methods that are limited in their sensitivity, and conventional molecular approaches that are limited in their scalability. In recent years the number of studies using NGS to investigate the microbial diversity and composition of ticks has expanded. Here, we provide a review of NGS strategies for tick microbiome studies and discuss the recent findings from tick NGS investigations, including the bacterial diversity and composition, influential factors, and implications of the tick microbiome.

## Background

A microbiome, or microbiota, can be defined as a community of commensal, symbiotic, and pathogenic microorganisms [1–3]. The tick microbiome consists of communities of viruses, bacteria and eukaryotes [4], and tick-borne pathogens (TBPs) of humans and animals are among the most important microorganisms that have been characterised within these arthropod vectors. Such pathogens of medical and veterinary importance include Crimean-Congo haemorrhagic fever virus (CCHFV), Kyasanur Forest disease virus (KFDV), and species of *Anaplasma*, *Borrelia*, *Coxiella*, *Ehrlichia*, *Francisella*, *Rickettsia*, *Babesia* and *Theileria* [5–7]. Importantly, however, the most dominant microorganisms that reside in ticks are obligate endosymbionts [8–10].

As microbiomes are typically composed of hundreds of microbial species [11, 12], laboratory methods that are capable of identifying multiple species simultaneously in a single assay offer great advantages. The development of next-generation sequencing (NGS) technologies in the last decade has enabled the genes and genomes of single cells and communities of microorganisms to be explored in a massively parallel, rapid, and cost-effective manner. Following the first NGS study of a tick microbiome in 2011 [13], the number of studies that have used NGS to investigate tick microbiomes has expanded (Table 1). Despite the growing number of studies, the microbiomes of most tick species remain unexplored, and further research into the functional role of these microorganisms in ticks at an individual and community level is needed.

**Table 1:** Summary of NGS studies that have described the diversity and composition of tick microbiomes

Location of study	Tick collection location	NGS platform	Target	Tick species	Host-collected or host-seeking	Tissues and instars	Reference
Amplicon sequencing							
Australia	New South Wales, Queensland, Victoria and Western Australia, Australia	MiSeq (Illumina)	16S (V1-V2)	*Ixodes holocyclus* (paralysis tick); *Amblyomma triguttatum* (kangaroo tick); *Haemaphysalis bancrofti* (wallaby tick); *Haemaphysalis longicornis* (bush or scrub tick)	Humans (*Homo sapiens*)	Whole adults and nymphs	[65]
	New South Wales, Australia; Freising and Leipzig, Germany	Ion Torrent (Thermo Fisher)	16S (V1-V2)	*I. holocyclus*; *Ixodes ricinus* (castor bean, deer or sheep tick)	Questing; cattle (*Bos taurus*); dogs (*Canis lupus familiaris*); echidnas (*Tachyglossus aculeatus*); cats (*Felis catus*); *H. sapiens*; kangaroos and wallabies (*Macropus* spp.); common brushtail possum (*Trichosurus vulpecula*); Australian raven (*Corvus coronoides*); Australian magpie (*Cracticus tibicen*)	Whole adults and nymphs	[50]
Eurasia	Mudanjiang, China	MiSeq (Illumina)	16S (V4)	*Ixodes persulcatus* (Taiga tick)	Questing	Whole adults	[52]
	Kibbutz Hulda, Israel	454 (Roche)	16S (V4-V6)	*Rhipicephalus turanicus*	Questing; *C. l. familiaris*	Whole adults	[51]
	Northern Italy	454 (Roche)	16S (V6)	*I. ricinus*	Questing	Whole adults and nymphs	[44]
	Shizuoka Prefecture, Japan	454 (Roche)	16S (V1-V3)	*Ixodes ovatus*; *I. persulcatus*; *Haemaphysalis flava*	Questing	Adult salivary glands	[40]
	Perak, Malaysia	Ion Torrent (Thermo Fisher)	16S (V6)	*Haemaphysalis wellingtoni*; *Haemaphysalis hystricis*; *Haemaphysalis bispinosa*	*C. l. familiaris*; *F. catus*; chickens (*Gallus gallus domesticus*)	Whole adults, nymphs and larvae	[48]
	Amasya and Sivas, Turkey	454 (Roche)	16S (V1-V3)	*Dermacentor marginatus*; *Rhipicephalus annulatus* (Blue cattle tick, cattle fever tick or Texas cattle fever tick)	*H. sapiens*	Whole adults	[132]
North America	Atlantic Canada and Ontario, Canada	Ion Torrent (Thermo Fisher)	16S (V1–2, V2, V3, V4, V5-V6, V6-V7, V8, V9)	*Ixodes scapularis* (deer or black-legged tick); *Ixodes angustus*	Questing; deer (Cervidae sp.); *C. l. familiaris*	Whole females and nymphs	[36]
	California, USA	MiSeq (Illumina)	16S (V4)	*Dermacentor occidentalis* (Pacific coast tick)	Questing	Whole adults^a^	[45]
	Connecticut, New York, North Carolina, South Carolina and Virginia, USA	454 (Roche) and MiSeq (Illumina)	16S (V1-V3, V4)	*I. scapularis*; *Ixodes affinis*	Questing	Whole adults	[41]
	Georgia, New York and North Carolina, USA	454 (Roche)	16S (V3-V5)	*Amblyomma americanum* (lone star tick)	Questing	Whole adults and nymphs	[46]
	Idaho, USA^b^	454 (Roche)	16S (V4)	*Dermacentor andersoni* (Rocky Mountain wood tick)	Laboratory-reared colony; *B. taurus*	Salivary glands and midguts of adults, nymphs and larvae	[75]
	Indiana, USA	454 (Roche)	16S (V1-V3)	*Dermacentor variabilis* (American dog or wood tick); *I. scapularis*	Rodents (*Peromyscus leucopus* and *Microtus ochrogaster*)	Whole nymphs and larvae	[42]
	Indiana, USA	454 (Roche)	16S (V1-V3)	*D. variabilis*; *I. scapularis*	*P. leucopus*	Whole nymphs and larvae	[43]
	Louisiana, USA	454 (Roche)	16S (V1-V3)	*Amblyomma longirostre*; *Amblyomma nodosum*; *Amblyomma maculatum* (Gulf coast tick); *Haemaphysalis juxtakochi*	Wood thrush (*Hylocichla mustelina*); hooded warbler (*Wilsonia citrina*); indigo bunting (*Passerina cyane*a); worm-eating warbler (*Helmitheros vermivorum*)	Whole nymphs and larvae	[133]
	Mississippi, USA	454 (Roche)	16S (V1-V3)	*Amblyomma maculatum*	Questing; rabbits (*Oryctolagus cuniculus*); sheep (*Ovis aries*)	Adult midguts, salivary glands and saliva	[53]
	Mississippi, USA	454 (Roche)	16S (V3-V7)	*Amblyomma tuberculatum* (gopher tortoise tick)	Gopher tortoise (*Gopherus polyphemus*)	Whole females and midguts from females	[80]
	Montana and Oregon, USA	PacBio (Pacific Biosciences)	16S (V1-V9)	*D. andersoni*	Questing	Midguts and salivary glands from males	[17]
	New York, USA	MiSeq (Illumina)	16S (V3-V4)	*I. scapularis*	Questing; white-tailed deer (*Odocoileus virginianus*)	Whole larvae; midguts and salivary glands from females	[81]
	North Carolina, USA	454 (Roche)	16S (V1-V3)	*A. americanum*	Questing	Whole adults and nymphs	[54]
	Oklahoma, USA^b^	454 (Roche)	16S (V4-V6)	*A. maculatum*	Laboratory-reared colony; *O. aries*	Midguts and salivary glands from adults	[82]
	Oklahoma, USA^b^	MiSeq (Illumina)	16S (V3-V4)	*A. americanum*	Laboratory-reared colony	Whole females	[77]
	Tennessee, USA	MiSeq (Illumina)	16S (V1-V4)	*A. americanum*	Questing	Whole adults^a^	[47]
	Texas, USA	454 (Roche)	16S (V1-V3)	*Rhipicephalus* (*Boophilus*) *microplus* (cattle tick)	*B. taurus*	Whole adults and eggs; midguts and ovaries from females	[13]
	Texas, USA^b^	Ion Torrent (Thermo Fisher)	16S (V5)	*A. americanum*	Laboratory-reared colony; *G. g. domesticus*	Whole adults and nymphs	[71]
	USA^c^	454 (Roche)	16S (V2)	*I. scapularis*	Laboratory-reared colony; *Or. cuniculus*	Whole larvae; midguts from nymphs	[83]
	USA^c^	MiSeq (Illumina)	16S (V4)	*I. scapularis*	Laboratory-reared colony; mice (*Mus* sp.)	Midguts from nymphs	[108]
	Western USA	MiSeq (Illumina)	16S^d^	*Ixodes pacificus* (western blacklegged tick)	Questing; *Mus sp*.; lizards (*Sceloporus occidentalis*)	Whole adults, nymphs and larvae	[109]
Shotgun Sequencing							
Asia	Yunnan, China	Ion Torrent (Thermo Fisher)	Viral cDNA^e^	*Rhipicephalus* spp.	Questing	Whole adults	[97]
	Hubei, Zhejiang, Beijing and Xinjiang, China	HiSeq (Illumina)	Viral cDNA^e^	*D. marginatus*; *Dermacentor* spp.; *Haemaphysalis doenitzi*; *H. longicornis*; *Haemaphysalis formosensis*; *Hyalomma asiaticum*; *R. microplus*; *Argas miniatus* (fowl tick)	Questing; wild and domestic animals	Whole “individuals”	[98, 99]
	Miyazaki, Japan	454 (Roche)	Bacterial and archaeal DNA^f^	*Amblyomma variegatum* (tropical bont tick); *I. ovatus*, *I. persulcatus*; *I. ricinus*; *Amblyomma testudinarium*; *H. formosensis*; *H. longicornis*	Questing	Whole adults and nymphs	[22]
Europe	Alsace, France	HiSeq (Illumina)	Bacterial cDNA^g^	*I. ricinus*	Questing	Whole nymphs	[23]
North America	New York, USA	Ion Torrent (Thermo Fisher)	Viral cDNA^e^	*A. americanum*; *D. variabilis*; *I. scapularis*	Questing	Whole adults	[100]

^a^Tissues were homogenised for one-half of the tick

^b^Origin of laboratory colony

^c^Origin of laboratory colony not specified

^d^Hypervariable region not specified

^e^Virome sequencing

^f^Whole genome amplification used for target amplification

^g^Whole transcriptome analysis

This review compares the advantages and disadvantages associated with NGS platform selection and sequencing methods (amplicon and shotgun sequencing). The use of NGS data to characterise the tick microbiome through measures of diversity and composition is discussed and recent discoveries of bacteria that are pathogenic, symbiotic, novel and associated with tick organs are highlighted. We also consider aspects of the tick microbiome that are largely unexplored and factors that influence the structure and organisation of the microbiome.

## Microbiome next-generation sequencing strategies

Two types of NGS can be applied to investigate microbiomes, amplicon sequencing and shotgun sequencing (which includes metagenomics and transcriptomics). There are nine hypervariable regions (V1-V9) of the bacterial 16S ribosomal RNA gene (16S) that can be targeted to identify bacterial taxa in 16S amplicon NGS studies, and regions V1-V4 have been most commonly sequenced in ticks (Table 1). 16S amplicon sequencing in ticks has been performed with 454 (Roche) pyrosequencing, Ion Torrent (Thermo Fisher) sequencing by semiconductor ion detection, and MiSeq (Illumina) platforms that use fluorescent dye detection sequencing methods (Table 1). Most of the published bacterial microbiome studies on ticks have used 454 platforms, such as the 454 GS Junior + and 454 GS FLX Titanium XL+, which have the advantage of longer read lengths (up to 1 kbp) compared to the Ion Torrent and MiSeq platforms [14], however, these sequencers have been discontinued. The longest read lengths achieved by Illumina platforms are up to 600 bp (single-end reads; 300 bp paired-end reads) on the MiSeq (with v3 chemistry) [15], and read lengths of up to 400 bp (single-end reads) can be sequenced on the Ion Torrent platforms Ion PGM and Ion S5 [16]. Greater 16S read lengths can improve taxonomic resolution of the sequences, and although almost full length 16S sequencing has been achieved on other platforms such as PacBio (Pacific Biosciences) [17], the cost of sequencing long amplicons is considerably more, and longer reads are provided at the expense of output read number [18], which is an equally important consideration for microbiome studies. The output read number is important for capturing adequate microbiome diversity, and the read numbers provided by the MiSeq are much greater than the Ion Torrent platform with comparable maximum read lengths; the MiSeq is capable of up to 50 million paired-end reads [15], whereas the Ion S5 530 platform can achieve up to 20 million single-end reads [16]. For shotgun sequencing, the HiSeq X (Illumina) sequences 150 bp paired-end reads and has the highest read output (2.6–3 billion reads) and lowest cost of sequencing (US$7 per Gbp) compared to other shotgun sequencing platforms [18, 19]. A large number of output reads is important in shotgun sequencing for better metagenome (or transcriptome) coverage. PacBio [20] and MinION (Oxford Nanopore) [21] are single-molecule long-read sequencers, with read lengths of ~20 kbp and 200 kbp, respectively, that can be used for metagenomics. Although the output number of reads are low on these platforms (< 350,000 sequences), superior genome coverage is afforded by greater read lengths; however, these platforms are considerably more expensive than short-read sequencing technologies [18]. At present, only short-read sequencers have been used for shotgun sequencing of tick metagenomes, transcriptomes and viromes (Table 1).

**Fig. 1:**
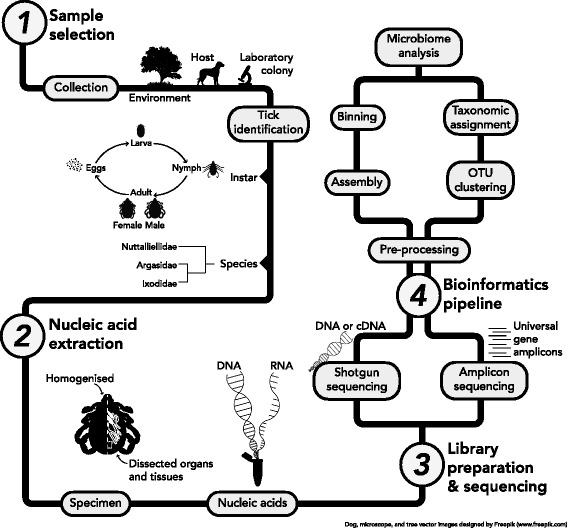
Schematic of the NGS workflow for studying the tick microbiome

Shotgun sequencing offers some advantages over amplicon sequencing such as the assessment of whole or partial genomes, transcriptomes, or viromes of the microbiome [22], and the sequencing of genes that are transcribed in microbes implies that they are actively replicating within the tick [23]. Furthermore, library preparation kits that are PCR-free can be used for shotgun sequencing library preparation [24], which eliminates the issues of PCR bias that amplicon sequencing is subjected to. However, at the current time, shotgun sequencing is considerably more expensive than amplicon sequencing [18], which may explain why the latter has been more widely used. Also, the nucleic acid extraction procedures for shotgun sequencing are more complex and require additional purification or enrichment steps compared to genomic DNA (gDNA) extraction methods for 16S amplicon sequencing. Purification or enrichment of microbial DNA needs to be performed for shotgun sequencing of viral and microbial metagenomes, as well as metatranscriptomes, to increase the genome, virome, or transcriptome coverage by reducing eukaryotic DNA from the tick and from the tick’s host [25–28]. Shotgun sequencing also results in significantly larger datasets than amplicon sequencing, which requires much more powerful computational tools for data storage and bioinformatic analyses. The approaches and software packages for NGS bioinformatics have been reviewed elsewhere [19, 29]. A schematic of the workflow for tick microbiome NGS studies is outlined in Fig. 1.

## Bacterial diversity

The diversity of the microbiome can be assessed through measures of alpha and beta diversity; alpha diversity measures the number of species in a sample and their proportion (species richness), while beta diversity measures the dissimilarity between samples (genetic relatedness) [30–32]. Diversity metrics depend on the taxonomic resolution of sequences and sequencing depth [33]. 16S NGS of one to three hypervariable regions results in read lengths of ~200–500 bp, which is a sufficient length for the taxonomic resolution of many, but not all, bacterial species. The nine hypervariable regions of 16S that enable bacterial taxa to be identified exhibit varying degrees of sequence diversity, and unfortunately, no single hypervariable region can be used to distinguish between all bacterial species [34, 35]. Although regions V1-V4 have been most commonly targeted in tick microbiome studies, a recent study that compared the bacterial diversity obtained from sequencing regions V1-V9 on the Ion Torrent found that regions V2, V3, V4, V6-V7, V8 and V9 gave the most comprehensive estimates of bacterial families, and the V4 region resulted in the highest estimated diversity [36]. Moreover, within some bacterial genera, the hypervariable regions of 16S are highly conserved between species, which limits species-level identification, e.g. the genus *Rickettsia*, especially the spotted fever group rickettsiae (SFGR) [37]. Additionally, the choice of similarity cut-offs and clustering algorithms used to pick operational taxonomic units (OTUs) [38, 39] and sequencing error rates [33] can also affect taxonomic resolution.

Obtaining a sufficient number of reads per sample (referred to as depth, or coverage, in amplicon NGS studies) is required for adequate measurements of diversity [33]. Alpha diversity rarefaction plots, which compare the number of OTUs obtained to sequencing depth, should be assessed to determine whether adequate sequencing depth was achieved for each sample, and these plots can be produced using Quantitative Insights Into Microbial Ecology (QIIME) software. A plateau in the plot suggests that adequate depth was achieved, whereas increasing trends suggest that further sequencing depth is required. Since the microbial diversity can vary in ticks based on factors such as species [40–43] and geographical location [41, 44–47], the required sequencing depth differs between studies. For example, the alpha diversity rarefaction plots from the study by Rynkiewicz et al. [43] plateau at ~20,000 reads for *Ixodes scapularis* (deer or black-legged tick) and *Dermacentor variabilis* (American dog or wood tick), whereas the alpha diversity rarefaction plots for most samples in the study by Khoo et al. [48] have an increasing trend up to 40,000 reads for *Haemaphysalis* spp. However, under-estimation of diversity can result when highly abundant sequences are overrepresented, regardless of sequencing depth. In such situations, PCR may not be sufficiently sensitive to amplify less abundant species. Blocking primers, which are primers that are modified with a DNA oligonucleotide or clamping probe that do not prime amplification, can be designed to reduce the amount of amplification of abundant template DNA during PCR [49]. This approach in *Ixodes* spp. has been shown to result in a significant increase in the total detectable bacterial diversity [50].

## Bacterial composition

The bacterial composition, or relative abundance of the taxa identified, refers to the proportion of reads for each taxon relative to the total number of reads for all taxa and can be assessed for each individual sample or for a particular variable (e.g. tick species, collection locality, life stage, host). The most abundant bacterial species that have been identified frequently in ticks include endosymbionts such as *Coxiella* spp. [13, 40, 51], *Rickettsia* spp. [42, 44, 51–54], *Francisella* spp. [42, 53] and “*Candidatus* Midichloria mitochondrii” [50]. However, pathogenic, environmental and skin-associated bacteria have also been reported as highly abundant [13, 44]*.* The number of reads obtained for each bacterial species are not necessarily representative of the number of microorganisms that were present in the tick due to the multi-copy nature of 16S [55]. Estimates of the number of bacterial species initially present could be made by correcting for the number of 16S copies in each species, or by targeting a single copy gene (e.g. *rpoB* gene). However, PCR amplification bias occurs when the amplification efficiency of templates differ, which leads to over- or under-representation of template DNA sequences [56, 57], and this can impact diversity measures of the dataset [38]. PCR conditions can be optimised to reduce, but not eliminate, the levels of PCR bias, such as reducing temperature ramping speeds, extending the denaturation step to provide sufficient time for GC-rich DNA fragments to denature, adding betaine to keep GC-rich templates single-stranded and using different enzymes such as AccuPrime Taq HiFi (Thermo Fisher) to improve priming specificity [58]. Alternative nucleic acid amplification methods exist, but due to the lack of commercial availability [59], PCR is the mainstay of amplicon NGS library preparation procedures.

## The bacterial microbiome of ticks

### Bacterial pathogens

Some of the most important bacterial TBPs include species of *Anaplasma*, *Borrelia*, *Ehrlichia*, *Francisella* and *Rickettsia*. *Ixodes ricinus*, the castor bean (or deer or sheep) tick, is prevalent throughout Europe and is responsible for transmitting a range of bacterial pathogens. Due to its significant role as a bacterial vector, *I. ricinus* has been commonly investigated using 16S NGS [22, 23, 44, 50]. *Ixodes ricinus* ticks sampled from Lyme disease endemic regions were found to carry pathogenic species within the *Borrelia burgdorferi* (*sensu lato*) (*s.l*.) genogroup, including *B. burgdorferi* (*sensu stricto*) (*s.s*.), *Borrelia garinii* and *Borrelia afzelii* [23, 44, 50]; as well as *Anaplasma phagocytophilum*, *Rickettsia helvetica* and “*Candidatus* Neoehrlichia mikurensis” [23, 60]. Known TBPs are usually identified at species-specific loci with sensitive qPCR or multiplex PCR screening procedures that are faster and more affordable compared to NGS in diagnostic settings, i.e. when a tick has been removed from a patient with clinical illness [61]. Since 16S NGS can be harnessed as a broad screening tool for bacteria, it is a useful technique for non-hypothesis driven surveillance of bacterial TBPs. Although greater sensitivity with qPCR compared to NGS has been demonstrated in some studies [62, 63], other studies have shown that increasing the sequencing depth can increase the chances of detecting rare species [64].

Sequencing of universal genes with NGS also allows for novel species identification [50, 65]. Determining the pathogenicity of newly discovered bacteria is then challenging as the direct association between a particular clinical illness and the bacterial species, once within the vertebrate host, must be proven. To a limited extent, the potential for pathogenicity of novel species can be estimated at the molecular level, e.g. by how they cluster genetically with known TBPs and by the presence of ‘pathogenicity islands’ that code for virulence genes [66, 67]. Paradoxically, however, pathogenicity islands have also been found in non-pathogenic species [68]. The identification of novel microorganisms in ticks using NGS may lead in time to an increase in what has been termed ‘reversed discovery of disease’, whereby the microorganism is identified in ticks before its pathogenicity to animals or humans is established [69].

### Endosymbiotic bacteria

Endosymbiotic bacteria that reside in hard ticks (ixodids) include species of *Coxiella*, *Francisella*, *Rickettsia* and “*Candidatus* Midichloria mitochondrii” [70]. *Coxiella*-like endosymbionts (CLEs) have been found in a variety of ixodids using NGS, including *Amblyomma americanum* (lone star tick) [71], *Haemaphysalis flava* [40], *Ixodes ovatus* [40], *Rhipicephalus* (*Boophilus*) *microplus* (cattle tick) [13], *Rhipicephalus turanicus* [51] and *Rhipicephalus sanguineus* (*sensu lato*) (*s.l.*) (brown dog tick) [51], and have also been found in soft tick (argasid) species, e.g. *Ornithodoros* spp. [60, 72]. Andreotti et al. [13] found that a CLE was most abundant in female *R. microplus* ovaries. Transovarial transmission of this CLE was recently demonstrated in *Ornithodoros maritimus*, *A. americanum, R. sanguineus* (*s.l.*) and *R. microplus* [72]. Although these CLEs purportedly have a symbiotic relationship with their tick hosts, a recent study by Angelakis et al. [73] detected a CLE from *Rhipicephalus* spp. (“*Candidatus* Coxiella massiliensis”) in skin biopsies from people with clinical signs of fever, skin eschar and local lymph node enlargement, and this organism is now therefore linked with human infection [73]. The causative agent of Q fever in humans, *Coxiella burnetii*, is a relatively recent descendant of CLEs of ticks [72], and seven tick species have been experimentally demonstrated as competent vectors of *C. burnetii* [74].

Endosymbionts are an important component of the microbiome that can benefit tick survival. Rickettsial endosymbionts of *Dermacentor andersoni* (Rocky Mountain wood tick) and *A. americanum*, and the CLE of *A. americanum* are examples of endosymbionts that are essential for the survival and reproductive fitness of their host. Studies that exposed *D. andersoni* and *A. americanum* to antibiotics found that the exposed colony’s progeny had lower numbers of their respective endosymbionts, which reduced the survival, feeding and moulting competence in *D. andersoni* [75], and reduced fecundity in *A. americanum* [75, 76]. Although a complete understanding of the mechanisms that cause endosymbionts to promote tick survival and fecundity is lacking, endosymbionts may play a key role in providing essential nutrients and cofactors that are absent from the blood meal. For example, Smith et al. [77] sequenced the genome of the CLE of *A. americanum* to investigate the potential for nutrient-provisioning, and found that the genome encodes for major vitamin and cofactor biosynthesis pathways [77].

### Environmental and skin-associated bacteria

It remains uncertain whether ubiquitous bacteria associated with soil, plants and skin that are frequently reported in NGS studies of ticks are contaminants from environmental or host sources, or whether they are genuinely associated with the tick microbiome. The tick’s exoskeleton can be sterilised with bleach prior to nucleic acid extraction in an attempt to remove contaminant DNA, or bioinformatics pipelines can be used to remove contaminating reads that are present in extraction and no-template controls [23]. Studies that have used sterilisation techniques (e.g. washing the ticks with 10% sodium hypochlorite) have, however, still detected environmental and skin bacteria in ticks [44, 50, 78]. This may be due to inadequate sterilisation (i.e. bacteria may remain hidden in crevices that are not exposed to the bleach solution during washing), or these bacteria may be ingested by ticks during feeding, therefore may be present in the tick midgut. As ticks spend the majority of their lives in the environment, saprophytic bacteria may be acquired at some point during their life cycle.

Bacterial contamination is a widespread issue among NGS datasets due to the sensitivity of this technology [79]. Stringent laboratory procedures must be followed during sample handling to minimise laboratory-derived contamination; the use of sterile gloves, workstations, laminar flow hoods, PCR grade water and pipette tips with filters are some examples of laboratory practices that can reduce contamination. Library preparation reagents can also be a source of contamination, e.g. ligases, polymerases and nucleotides are purified from bacteria, and *Bradyrhizobium* spp. have been reported in ultrapure water systems [79]. To ensure that these contaminants are controlled for, extraction and no-template controls should be included so that the contaminant sequences can be identified and removed bioinformatically from the dataset.

### Organ-associated bacteria

While most 16S NGS studies of ticks have extracted DNA from entirely homogenised ticks (Table 1), some researchers have examined bacterial profiles of tick organs [13, 40, 53, 80, 81]. Microbial communities differ between anatomical regions within the tick, such as the midgut, reproductive tract and the salivary glands [13, 17, 53, 75, 80–83]. Most TBPs are transmitted to the vertebrate host via salivary secretions during blood feeding. An assessment with 16S NGS of the bacteria present in the salivary glands of *I. ovatus*, *Ixodes persulcatus* (Taiga tick) and *H. flava* collected in Japan revealed a surprisingly large number of bacterial genera: 71 (*I. ovatus*), 127 (*I. persulcatus*) and 59 (*H. flava*) [40], and some of the medically important genera that were detected included *Coxiella*, *Ehrlichia* and *Rickettsia* [40].

*Rickettsia parkeri*, one of the causes of human rickettsiosis [84], is transmitted by *Amblyomma maculatum* (Gulf Coast tick), and has been detected in the midguts, salivary glands and saliva of questing ticks with NGS [53]. The study suggested that the bacteria may migrate from the midgut to the salivary glands prior to feeding [53], which differs from the transmission route of other pathogens, such as *B. burgdorferi*, which develops in the tick midgut of *Ixodes* spp. then translocates to the salivary glands during feeding [85, 86].

It is possible that dissected tick organs may be contaminated with microbes from surrounding tissues or adjacent organs. Methodologies to control for this should be implemented, otherwise alternative assays may need to be utilised e.g. fluorescence in situ hybridization (FISH) [87]. Immunofluorescence assays have provided insights into the transstadial transmission of a novel relapsing fever *Borrelia* sp. found in *Amblyomma geoemydae* in Japan, which was visualised in the midgut and salivary glands of instars after moulting [88].

## Neglected facets of the tick microbiome

### Viruses

Viral species transmitted by ticks include tick-borne encephalitis virus [89], CCHFV [90], KFDV [91], Alkhurma virus, severe fever with thrombocytopenia syndrome virus (SFTSV) [92], Heartland virus (HRTV) [93], Powassan and Colorado tick fever viruses [94, 95], Africa swine fever virus, Nairobi sheep disease virus (NSDV) and Louping ill virus [5, 6]. Shotgun sequencing of viral cDNA is a more comprehensive approach than amplicon sequencing for studying the tick virome due to the lack of universal genes in viruses [96]. Thus far, only four shotgun NGS studies have investigated the virome of ticks [97–100]. Xia et al. [97] sequenced the virome of *Rhipicephalus* spp. using the Ion Torrent platform in China and discovered novel anellovirus and nairovirus species. The most abundant sequences were nairoviruses, phages and invertebrate viruses [97]. Most of the recognised nairoviruses are transmitted by ticks, and some that impact human and animal health, such as CCHFV and NSDV, have been identified in *Rhipicephalus* spp. previously [101]. Tokarz et al. [100] discovered eight previously uncharacterised viral sequences in *I. scapularis*, *D. variabilis* and *A. americanum* from the USA that were most similar to nairovirus, phlebovirus, invertebrate-like virus, mononegavirus and tetravirus-like virus, and also identified Powassan virus in *I. scapularis* [100]. Li et al. [98] and Shi et al. [99] investigated the viromes of a wide variety of invertebrates, including ticks from China, and their results suggest that arthropods are major reservoir hosts for negative-sense RNA viruses that are found in vertebrates, plants, fungi and protists. The considerable number of known tick-borne viruses and the growing number of discoveries of novel viruses potentially transmitted by ticks warrants a wider investigation of tick viromes.

### Eukaryotes

Protozoan tick-borne diseases (TBDs), such as babesiosis and theileriosis, are caused by the piroplasms *Babesia* spp. and *Theileria* spp., respectively [102]. To date, no 18S ribosomal RNA gene (18S) NGS studies on ticks have been published, and there have been no protozoan discoveries reported in shotgun sequencing studies on ticks. Shotgun sequencing could be used to identify protists in addition to viruses and bacteria; however, the relative abundance of eukaryotic tick DNA is much greater than protist DNA, and so a great sequencing depth would be required for protist detection. 18S ranges from 1.5 kbp to more than 4.5 kbp [103], and like bacterial 16S, 18S contains nine hypervariable regions (V1-V9). The use of universal 18S primers for identifying eukaryotic DNA with amplicon sequencing in ticks is challenging as other eukaryotic species from soil flora contaminants, animals and the tick itself will also be amplified. It may be possible to address this issue with blocking primers [50], or the use of protist-specific primers for NGS [104]. In addition to *Babesia* and *Theileria,* other Eukarya that require further characterisation at the community level in ticks include other apicomplexan species (e.g. *Hepatozoon* spp.), trypanosomes [105], fungi [106] and helminths [107].

## Factors influencing the microbiome

Several studies have assessed the effect of environmental and host-related factors on tick microbiome diversity and composition. Environmental factors such as geographical location [41, 44–47], temperature, humidity [71], season [51], habitat type and soil type have been shown to influence the bacterial diversity and composition of ticks. Microbiome diversity and composition can also vary depending on vertebrate host and arthropod-related factors such as tick species [40–43], instar and sex [41, 44–47, 51, 54, 71, 81], anatomical location (e.g. midguts and salivary glands) [13, 53, 75, 81] and blood-feeding [52, 108, 109].

Other studies have assessed additional factors, such as the role of proteins in the microbiome that are encoded by the tick’s genome, and their effect on pathogen acquisition. Silencing of transcripts using RNA interference (RNAi) of the antioxidant selenoproteins thioredoxin reductase (TrxR) and glutathione peroxidase (GPx) reduced the bacterial load of Rickettsiaceae in *A. maculatum* midguts and salivary glands [82], and *B. burgdorferi* in *I. scapularis* saliva [110], respectively. RNAi-mediated knockdown of the selenocysteine-specific elongation factor (SEF) gene in *A. maculatum*, which is important for selenoprotein translation, resulted in no detectable *R. parkeri* in *A. maculatum* midguts [111]. Other protein-encoding genes also affect pathogen acquisition and development, and are differentially regulated in ticks in response to *Anaplasma marginale* infection [112].

NGS investigations of ticks have also revealed that microbe-microbe interactions can influence microbiome composition. For example, Gurfield et al. [45] found that there was an inverse relationship between the number of *Francisella*-like endosymbionts (FLEs) and SFGR in *Dermacentor occidentalis*, which suggests that FLEs have an ability to interfere with SFGR colonisation [45]. Other non-NGS studies have demonstrated that endosymbionts can interfere with TBP transmission by affecting pathogen acquisition and colonisation [70, 113, 114]. Narasimhan et al. [83] altered the gut microbiota of *I. scapularis* by rearing larvae in sterile containers, and 16S 454 sequencing showed that the dysbiosed larvae had a higher abundance of Proteobacteria, including the genera *Rickettsia*, *Thioclava* and *Delftia*, compared to control larvae reared under normal conditions. In that study, qPCR assessments of larvae fed on *B. burgdorferi*-infected mice and pathogen-free mice showed that *B. burgdorferi* colonisation in dysbiosed larvae, as well as gentamycin-exposed larvae, was significantly reduced compared to the control larvae [83].

Similarly, a study by Gall et al. [17] showed that higher proportions of the endosymbiont *Rickettsia bellii* in *D. andersoni* were correlated with decreased acquisition of the pathogen *A. marginale*. However, generalisations cannot be made about the effect of all types of endosymbionts on pathogen acquisition across various tick species. Conversely, it was shown that the pathogen acquisition of *Francisella novicida* was positively correlated with high proportions of FLEs in *D. andersoni* [17].

Pathogens can also influence the tick gut and its microbiota. In *I. scapularis*, the pathogen *A. phagocytophilum* induces the protein *I. scapularis* antifreeze glycoprotein (IAFGP) [115], and was shown in a study by Abraham et al. [108] to decrease the abundance of the genera *Enterococcus* and *Rickettsia* and increase *Pseudomonas* in *I. scapularis* midguts*.* The study by Abraham et al. [108] also showed that *A. phagocytophilum* decreased the expression levels of peritrophin-1, peritrophin-2 and peritrophin-4, which are genes that encode for peritrophin, a major component of peritrophic matrix (PM) glycoproteins that form a layer separating epithelial cells from the tick gut lumen, and this caused a decrease in the thickness of the PM. This study demonstrated that *A. phagocytophilum* induced changes in the gut barrier by decreasing peritrophin expression, which enhanced the colonisation of *A. phagocytophilum* in the tick midgut [108].

Macroparasites of ticks may also influence the microbiome. It has been suggested that *Wolbachia pipientis* may not be a naturally occurring endosymbiont of ticks, but rather has been introduced to the tick microbiome by a parasitic wasp [116]. Further studies are required to establish the extent to which environmental, tick and vertebrate host-related factors influence the microbiome. Other variables that could also be considered include degree of engorgement, gut protein expression responses to a blood meal [117, 118], host immune molecules present in blood meals [119], tick gut changes in response to feeding [120], tick copulation and egg fertilisation [120, 121], vertebrate host skin microflora, microorganisms in the vertebrate host’s blood and community changes brought about by microbial interactions within the tick.

## Implications of microbiome studies

Not only is NGS a comprehensive tool for studying tick microbiomes, it can also be used for TBP or ‘pathobiome’ surveillance to improve TBD diagnostics [122] and for the reversed discovery of human and animal TBDs. Importantly, an understanding of the factors that influence the microbiome, and the role of the microbiome, provides new avenues to be explored for TBD control. Most tick and TBD control strategies focus on the use of acaricides and vaccines [123–125]; however, tick populations can become resistant to chemical acaricides [123], and vaccine development takes an average of ten years [126]. Strategies could be developed to manipulate the tick microbiome to decrease the vectorial capacity of ticks by hindering pathogen acquisition, development, and horizontal and vertical transmission, which could have a long-term impact on TBP transmission and could ultimately reduce morbidity and mortality caused by TBDs. In the field of human microbiome research, investigations of metabolic, signaling and immune interactions between gut microbes and host physiology have led to the concept of therapeutic microbial manipulation to treat or prevent diseases [127–129]. For example, faecal microbiota transplantations have been successfully used to reestablish colonic microbial populations that fight against intestinal infections with *Clostridium difficile* [130].

Microbiome alterations have also been induced in arthropod vectors to combat vector-borne diseases. The endosymbiont *W. pipientis* causes cytoplasmic incompatibility and has been introduced into mosquito vectors such as *Culex pipiens*, *Aedes aegypti*, *Aedes albopictus* and *Aedes polynesiensis* to control their populations with the aim to reduce the transmission of diseases such as filariasis, dengue fever, yellow fever, chikungunya and Zika fever. There are ongoing trials and evaluations of releasing *Wolbachia*-infected *Ae. aegypti* in Brazil, Colombia, Indonesia, Singapore and Vietnam [131]. Similar microbial management strategies could be developed for ticks that promote the growth of endosymbiotic bacteria, such as *R. bellii*, to reduce acquisition of pathogens, such as *A. marginale* in *D. andersoni*, or strategies could be developed to the hinder the growth of FLEs in *D. andersoni* to reduce *F. novidica* acquisition [17]. Genes that could be manipulated to hinder pathogen acquisition and development include antioxidant selenoprotein genes and PM glycoprotein genes. Silencing of TrxR in *A. maculatum* and GPx in *I. scapularis* negatively affects *R*. *parkeri* and *B. burgdorferi* development, respectively [82, 110, 111]. Preventing *A. phagocytophilum* from decreasing the expression levels of peritrophin-1, peritrophin-2 and peritrophin-4 would impede *A. phagocytophilum* growth in the tick midgut [108].

## Conclusions

Next-generation sequencing methodology is a powerful technique that is revolutionising our understanding of TBPs, endosymbiotic bacteria and other microbes associated with the tick microbiome. Identification of species that form this arthropod’s microbiome is fundamental to exploring its functions, yet despite this, there is currently a dearth of published studies investigating viruses and eukarya in ticks. Additionally, as this research is still in its infancy, the microbiomes of many tick species remain to be investigated. The influences of the environment, the vertebrate host and the tick itself on the microbial diversity and composition in ticks need to be further defined and assessed. This is an important consideration for study design and will have an impact on the interpretation and biological relevance of the findings. Further tick microbiome research is required to increase our understanding of the molecular and biochemical basis of tick microbiome interactions, and as this improves, novel microbial management strategies for TBDs may be developed in the future.

## Abbreviations

16S: 16S ribosomal ribonucleic acid gene
18S: 18S ribosomal ribonucleic acid gene
CCHFV: Crimean-Congo haemorrhagic fever virus
cDNA: complementary DNA
CLE: *Coxiella*-like endosymbiont
FISH: fluorescence in situ hybridization
FLE: *Francisella*-like endosymbiont
Gbp: gigabase pairs
gDNA: genomic deoxyribonucleic acid
GPx: glutathione peroxidase
HRTV: Heartland virus
IAFGP: *Ixodes scapularis* antifreeze glycoprotein
Kbp: kilobase pairs
KFDV: Kyasanur Forest disease virus
NGS: next-generation sequencing
NSDV: Nairobi sheep disease virus
OTU: operational taxonomic unit
PM: peritrophic matrix
QIIME: Quantitative Insights Into Microbial Ecology
qPCR: real-time polymerase chain reaction
RNAi: ribonucleic acid interference
SEF: selenocysteine-specific elongation factor
SFGR: spotted fever group rickettsiae
SFTSV: severe fever with thrombocytopenia syndrome virus
TBD: tick-borne disease
TBP: tick-borne pathogen
TrxR: thioredoxin reductase
V1-V9: hypervariable regions 1–9
